# Predictors of the Diabetes-Specific Quality of Life in Adolescents With Type 1 Diabetes Mellitus: A Hospital-Based Cross-Sectional Study

**DOI:** 10.7759/cureus.104475

**Published:** 2026-03-01

**Authors:** Seena Bhageerathy, Anita David, Geetha Saradakutty, Saji James, Riaz Ismail

**Affiliations:** 1 Nursing, Government Medical College, Thiruvananthapuram, Thiruvananthapuram, IND; 2 Paediatric Nursing, Sri Ramachandra Institute of Higher Education and Research, Chennai, IND; 3 Paediatrics, Government Medical College, Thiruvananthapuram, Thiruvananthapuram, IND; 4 Pediatrics, Sri Ramachandra Institute of Higher Education and Research, Chennai, IND; 5 Pediatrics, Government Medical College, Thiruvananthapuram, Thiruvananthapuram, IND

**Keywords:** diabetes-specific quality of life, glycemic control, hba1c, high-performance liquid chromatography, type 1 diabetes mellitus

## Abstract

Introduction

Adolescence is a crucial developmental stage marked by significant changes in social, psychological, and physical aspects of life. The demands of managing type 1 diabetes mellitus (T1DM) throughout life, such as administering insulin, monitoring blood sugar, controlling nutrition, and fear of both acute and long-term complications, exacerbate these developmental difficulties for teenagers. Their quality of life (QoL) can be significantly impacted by these factors; hence, evaluating QoL and its determinants is a crucial part of comprehensive diabetes management.

Methods

An analytical cross-sectional study was conducted at the Diabetic Clinic of Government Medical College, Thiruvananthapuram, a tertiary care center in Kerala, India, with 141 adolescents aged 10 to 18 years who attended the diabetic clinic from July 2023 to February 2024. All were on regular treatment for at least six months. Data collection utilized a structured questionnaire that included both sociodemographic and diabetes-related data. HbA1c was measured using high-performance liquid chromatography (HPLC). Based on the International Society for Pediatric and Adolescent Diabetes (ISPAD) 2022 criteria, children were classified as having good (<7%) or poor (>7%) glycemic control. Diabetes-specific QoL (DSQoL) was measured by using the PedsQL 3.2 Diabetes Module.

Results

Among the 141 participants, 83 participants (58.9%) had a DSQoL score of less than 70, indicating that more than half of the adolescents with T1DM had impaired QoL. Of the adolescents with diabetes, only 41.1% showed optimal QoL. This result shows that adolescents have a significant burden of physical, psychological, and treatment-related difficulties due to diabetes, which emphasizes that medical management alone is not enough, and psychosocial interventions are clearly essential. Factors including age, occupation of father, duration of diabetes, dietary adherence, physical activity, and glycemic control were found to be associated with DSQoL. Binary logistic regression identified independent predictors: age (adjusted odds ratio (aOR) = 2.703; 95% confidence interval (CI): 1.186-6.156; *p* = 0.018), occupation of father (aOR = 2.383; 95% CI: 1.040-5.459; *p* = 0.040), and glycemic control (aOR = 0.433; 95% CI: 0.192-0.973; *p* = 0.043).

Conclusion

The findings highlight that DSQoL is compromised in the majority of adolescents with T1DM. This underscores the need for targeted psychosocial interventions, improved self-management support, and regular assessment of QoL as part of routine diabetes care.

## Introduction

Type 1 diabetes mellitus (T1DM) is one of the most common chronic endocrine disorders affecting children and adolescents globally, with a substantial rise in low- and middle-income nations [1].

Lifelong insulin therapy, regular blood glucose monitoring, nutritional control, and lifestyle changes are all necessary for managing T1DM, which places a significant physical and psychological burden on affected adolescents and their families [2].

For those with T1DM, adolescence is a particularly vulnerable time because the complicated demands of managing the disease coincide with rapid somatic growth and pubertal maturation, emotional changes, and a growing need for independence. These difficulties frequently impact metabolic management and medication compliance, which in turn affects general well-being. As a result, in addition to more conventional clinical indications like HbA1c, quality of life (QoL) has become a significant patient-centered outcome in the treatment of pediatric diabetes [3].

The distinctive symptom load, treatment requirements, and disease-related issues associated with T1DM are captured by diabetes-specific QoL (DSQoL) tools. Adolescents with T1DM often have a lower QoL because of diabetes-related symptoms, treatment complexity, fear of hypoglycemia, and dietary restrictions, which are better measured by validated tools like the Pediatric Quality of Life Inventory^TM^ (PedsQL^TM^) Diabetes Module [3,4].

Age, glycemic control, duration of diabetes, dietary adherence, physical activity, and socioeconomic characteristics have all been found to be significant contributors to DSQoL in a number of worldwide and Indian studies [5,6]. While younger age and socioeconomic disadvantage are related to worse outcomes, better metabolic control and supportive family situations are linked to increased QoL. Studies conducted in India have also found that children and adolescents with T1DM have moderate to poor QoL, which can be attributed to issues with access to healthcare, the cost of diabetes treatment, and the lack of psychosocial support. Adolescents with diabetes showed a substantial correlation between their QoL and metabolic management, as determined by their HbA1c score. An improved QoL is linked to good glycemic management [7].

There is still limited data on DSQoL and its determinants among adolescents in an Indian setting, despite the growing focus on QoL measurement. Thus, the current study aimed to determine the overall and domain-wise DSQoL and its predictors among adolescents with T1DM.

## Materials and methods

An analytical cross-sectional study was conducted to identify the factors linked to DSQoL in adolescents with T1DM. The study was conducted at the Diabetic Clinic of Government Medical College, Thiruvananthapuram, a tertiary care center in Kerala, India, with 141 adolescents aged 10 to 18 years who attended the diabetic clinic, which serves as a referral center for T1DM management across three districts, namely, Thiruvananthapuram, Kollam, and Pathanamthitta, from July 2023 to February 2024. The sample size was determined based on previously reported mean DSQoL scores (mean ≈ 70, SD ≈ 15) using the PedsQL™ Diabetes Module; the sample size was calculated using a 95% confidence level and an absolute precision of 2.5 points [8]. The Institutional Ethics Committee of Government College of Nursing, Medical College, Thiruvananthapuram (Ref. No.: IEC-NI/19/NOV/71/87 dtd 09/12/2019) has granted ethical approval for the study. Written consent and assent were obtained from the parents and their adolescents after providing information sheets. Adolescents with T1DM eligible for the study were aged between 10 and 18 years and were on regular treatment for at least six months. Those with secondary diabetes or type 2 diabetes mellitus (T2DM) were excluded.

Participants were selected consecutively based on the established eligibility criteria. Data collection utilized a structured questionnaire that included both sociodemographic details (such as age, gender, place of residence, family type, parents' ages and education levels, parental occupations, and family income) and diabetes-related information (age of diagnosis, duration of diabetes, family history of T1DM, history of thyroid issues, type and frequency of insulin administration, weekly blood glucose monitoring frequency, dietary adherence by reviewing food diaries, weekly physical activity (vigorous and muscle strengthening activities for 45 minutes to one hour at least three days per week) and regularity of follow-up visits).

Glycemic control was assessed using high-performance liquid chromatography (HPLC), an analytical technique that measures HbA1c value. According to the International Society for Pediatric and Adolescent Diabetes (ISPAD) 2022 criteria, adolescents with HbA1c levels less than 7.0% and greater than 7.0% were categorized as having good glycemic control and poor glycemic control, respectively [9]. DSQoL was measured using the PedsQL 3.2 Diabetes Module (Self-Report). The study population completed the newly translated and validated regional language (Malayalam) version of the Pediatric Quality of Life Inventory (PedsQL) 3.2 Diabetes Module to assess DSQoL. The translation was completed in accordance with the linguistic validation requirements specified by the Mapi Research Trust [10]. Two independent professionals first translated the English questionnaire into the local language, Malayalam, and then generated a reconciled version. A different, non-medical translator who had not seen the original version backtranslated this to English. Following the approval by Mapi Research Trust, conceptual flaws were fixed, cognitive interviews were carried out, and the final version was produced and verified.

As per the Master User Licence Agreement signed between the study proponents and Mapi Research Trust, the developer of the original questionnaire holds the copyright of both the original and all translations of the questionnaire. 

The collected data were analyzed with IBM SPSS Statistics for Windows, Version 20.0 (released 2011, IBM Corp., Armonk, NY), which depicted DSQoL and the relations between the variables. The relationship between DSQoL and various sociodemographic and diabetes related variables was determined using Pearson’s Chi-square test, as applicable. Variables demonstrating association in the univariate analysis (p < 0.2) were further investigated using binary logistic regression to identify the independent predictors of DSQoL. A p-value of <0.05 was considered statistically significant.

## Results

The sociodemographic characteristics of the study participants revealed that the majority of adolescents were aged between 10 and 13 years (61%), with females constituting a slightly higher proportion (60.3%) than males. A considerable proportion of participants were residing in rural areas (76.6%), indicating that the sample predominantly represents a rural population, and the majority (68.1%) belonged to nuclear families.

Regarding parental characteristics, most fathers were aged below 45 years (55.3%). The educational status of the fathers was largely limited to higher secondary level (82.3%), and the majority of fathers were engaged in informal employment (68.8%), suggesting relatively lower occupational stability. Mothers were predominantly below 40 years of age (56.7%), with the majority having higher secondary education (73%), but only one-third of the mothers were employed (36.2%). Nearly half of the families reported a monthly income below ₹10,000 (48.9%), indicating a predominantly low socioeconomic background (Table 1).

**Table 1:** Sociodemographic characteristics of the study participants

Variables	Features	Frequency	Percentage
Age (in years)	10-13	86	61.0
	14-18	55	39.0
Gender	Female	85	60.3
	Male	56	39.7
Resident area	Urban	33	23.4
	Rural	108	76.6
Type of family	Nuclear	96	68.1
	Joint family	45	31.9
Age of father (in years)	<45	78	55.3
	>45	63	44.7
Education of father	Up to higher secondary	116	82.3
	Degree and above	25	17.7
Occupation of father	Informal employment	97	68.8
	Formal employment	44	31.2
Age of mother (in years)	<40	80	56.7
	>40	61	43.3
Education of mother	Higher secondary	103	73.0
	Degree and above	38	27.0
Occupation of mother	Home maker	90	63.8
	Employed	51	36.2
Monthly family income (in Rupees)	<10000	69	48.9
	>10000	72	51.1

T1DM was diagnosed at the age of seven to nine years in 42.6% of adolescents, followed by 10-12 years (27.0%), indicating that middle childhood was the most common period of diagnosis. Over half of the participants (51.8%) had a diabetes duration of one to three years, suggesting a relatively recent diagnosis in most cases. A positive family history ofT1DM (9.2%) and history of thyroid disorder (7.1%) were reported in a small proportion, highlighting limited clustering of autoimmune conditions within families.

Most children (81.6%) were on a basal-bolus insulin regimen (long-acting with short-acting insulin), and nearly half (48.9%) required five insulin injections per day, reflecting intensive insulin therapy practices. However, self-monitoring of blood glucose was suboptimal, with nearly two-thirds (65.2%) monitoring on fewer than three days per week.

Dietary adherence was reported by 70.9% of participants, indicating reasonably good compliance, while physical activity levels varied, with a notable proportion reporting either no activity (22.0%) or less than three days per week (41.1%). Although all participants were on regular follow-up, glycemic control remained suboptimal, with 62.4% having HbA1c levels above 7%. This suggests that despite structured treatment regimens and follow-up, significant gaps persist in self-management practices and achievement of optimal glycemic control (Table 2).

**Table 2:** Diabetes-related characteristics of the study participants

Variables		Frequency	Percentage
Age (in years) at diagnosis of TIDM	<3	16	11.3
	4-6	20	14.2
	7-9	60	42.6
	10-12	38	27.0
	>12	7	5.0
Duration of diabetes in years	1-3	73	51.8
	4-6	22	15.6
	7-9	30	21.3
	>9	16	11.3
Family history of TIDM	Yes	13	9.2
	No	128	90.8
History of thyroid disorder	Yes	10	7.1
	No	131	92.9
Type of insulin	Long-acting + short-acting	115	81.6
	Short-acting + intermediate-acting	26	18.4
Insulin frequency/day	3	17	12.1
	4	55	39.0
	5	69	48.9
Self-monitoring of blood glucose per week	<3 days	92	65.2
	>3 days	49	34.8
Dietary adherence	Yes	100	70.9
	No	41	29.1
Physical activity per week (in days)	No activity	31	22.0
	<3 days	58	41.1
	>3 days	83	58.9
Regular follow-up	Yes	141	100.0
HbA1c (%)	<7	53	37.6
	>7	88	62.4

More than half of the adolescents with T1D had impaired DSQoL as measured by the PedsQL Diabetes Module 3.2 (MAPI Research Trust), with 83 participants (58.9%) scoring below 70. Only 41.1% of the adolescents demonstrated optimal DSQoL. This finding indicates a substantial burden of diabetes-related physical, emotional, and treatment-related challenges among adolescents (Table 3).

**Table 3:** Proportion of the diabetis-specific quality of life among adolescents with type 1 diabetes mellitus (T1DM) PedsQL 3.2 Diabetes Module [4] Impaired QoL < 70 (Based on international norms) [4,8]

Diabetes-specific QoL	Frequency	Percentage
Impaired (PedsQL DM Score < 70)	83	58.9
Optimal (PedsQL DM Score > 70)	58	41.1

The domain-wise analysis of DSQoL as measured by the PedsQL Diabetes Module 3.2 (MAPI Research Trust) among adolescents with T1DM revealed moderately impaired overall QoL. The diabetes management domain showed the highest mean score (69.89 ± 13.45), indicating relatively better perceived competence and satisfaction with treatment-related activities such as insulin administration, glucose monitoring, and adherence to care routines. The diabetes symptoms domain had a comparatively lower mean score (63.58 ± 10.79), reflecting a greater burden of physical symptoms, glycemic fluctuations, and diabetes-related discomfort experienced by adolescents. Overall, the diabetes-specific total QoL score (66.60 ± 11.30) indicates a moderately impaired quality of life among adolescents with T1DM. The observed gap between management and symptom domain scores suggests that while adolescents may adapt reasonably well to treatment regimens, symptom burden remains a key challenge affecting their overall QoL(Table 4)

**Table 4:** Mean and standard deviation of domain-wise and overall diabetes-specific quality of life (DSQoL) among adolescents with type 1 diabetes mellitus (T1DM) PedsQL 3.2 Diabetes Module [4]

PedsQL Diabetes Module 3.2	N	Minimum	Maximum	Mean	SD
Diabetes symptom	141	32	90	63.58	10.79
Diabetes management	141	36	97	69.89	13.45
Diabetes-specific overall QoL	141	32	94	66.60	11.30

The present analysis demonstrated that several sociodemographic and diabetes-related factors were significantly associated with DSQoL. Age showed an association with QoL, with adolescents aged 14-18 years reporting better DSQoL compared to younger children aged 10-13 years (χ² = 8.64, p = 0.003). This may reflect improved cognitive maturity, coping skills, and autonomy in self-management among older adolescents.

Among parental characteristics, occupation of the father showed an association with DSQoL (χ² = 4.75, p = 0.03), with children of fathers in formal employment more likely to have optimal QoL. This finding suggests that job stability and better socioeconomic security may positively influence access to healthcare resources, treatment adherence, and psychosocial support. Other parental variables, including education and age of parents, were not associated with QoL.

Duration of diabetes was associated with diabetes-specific QoL (χ² = 5.22, p = 0.02), with children having a duration of less than five years showing impaired QoL compared to those with longer disease duration. This finding indicates that adaptation to chronic illness and the development of coping mechanisms improve over time.

Lifestyle and disease management factors showed strong associations with DSQoL. Dietary adherence (χ² = 6.69, p = 0.01), physical activity of more than three days per week (χ² = 7.47, p = 0.01), and good glycemic control (HbA1c <7%) (χ² = 6.47, p = 0.01) were found to be associated with optimal QoL. These results emphasized that effective self-management behaviors and metabolic control are critical determinants of perceived well-being in adolescents with T1DM. By contrast, gender, area of residence, monthly family income, family history of T1DM, insulin frequency, and frequency of self-monitoring of blood glucose did not show any association with DSQoL in this cohort (Table 5).

**Table 5:** Association of sociodemographic and diabetes related characteristics with diabetes-specific quality of life (DSQoL)

Variables		Diabetes-Specific QoL Score		X^2^	P
		<70 (Impaired)	>70 (Optimal)		
Age in years	10-13	59	27	8.64	0.003*
	14-18	24	31		
Gender	Male	32	24	0.11	0.74
	Female	51	34		
Area of Residence	Rural	61	47	1.08	0.29
	Urban	22	11		
Type of Family	Nuclear	53	43	1.66	0.19
	Joint family	30	15		
Age of Father (in years)	<45	50	28	1.98	0.16
	>45	33	30		
Education of Father	Upto Higher Secondary	68	48	0.02	0.89
	Degree and above	15	10		
Occupation of Father	Informal Employment	63	34	4.75	0.03*
	Formal Employment	20	24		
Age of mother( in years)	<40	52	28	2.87	0.09
	>40	31	30		
Education of mother	Upto Higher Secondary	59	44	0.39	0.53
	Degree and above	24	14		
Occupation of mother	Home maker	49	41	2.01	0.16
	Employed	34	17		
Monthly Family Income ( in Rupees)	<10000	38	31	0.80	0.37
	>10000	45	27		
Duration of Diabetes in years	<5	56	28	5.22	0.02*
	>5	27	30		
Family History of TIDM	Yes	08	05	0.04	0.84
	No	75	53		
Insulin frequency/day	3	12	05	1.13	0.57
	4	31	24		
	5	40	29		
Self monitoring of blood glucose per week	<3 days	54	38	0.003	0.96
	>3 days	29	20		
Dietary Adherence	Yes	52	48	6.69	0.01*
	No	31	10		
Physical activity per week(in days)	< 3 days	42	16	7.47	0.01*
	> 3 days	41	42		
HbA1c (%)	<7	24	29	6.47	0.01*
	>7	59	29		

Binary logistic regression analysis identified age of the child, occupation of the father, and glycemic control as independent predictors of DSQoL. Children aged 14-18 years were nearly 2.7 times more likely to have normal DSQoL compared to younger children (aOR = 2.703; 95% CI: 1.186-6.156; p = 0.018). This indicates that increasing age independently contributes to better QoL, possibly due to improved disease understanding, coping skills, and autonomy in self-care.

Similarly, father’s occupation showed a significant association with QoL. Children whose fathers were in formal employment had 2.38 times higher odds of reporting normal DSQoL compared to those with fathers in informal employment (aOR = 2.383; 95% CI: 1.040-5.459; p = 0.040). This underscores the role of socioeconomic stability in supporting effective diabetes management, access to healthcare resources, and psychosocial well-being.

Glycemic control also emerged as a significant protective predictor of QoL. Adolescents with good glycemic control have 56.7% lower odds of having poor DSQoL compared to those with poor control (aOR = 0.433; 95% CI: 0.192-0.973; p = 0.043), reinforcing the close link between metabolic control and perceived well-being (Table 6).

**Table 6:** Binary logistic regression among factors associated with diabetes-specific quality of life

Variables	aOR	95% CI		P
		Lower	Upper	
Age in years	2.70	1.19	6.16	0.02*
Type of family	0.69	0.30	1.59	0.39
Occupation of father	2.38	1.04	5.46	0.04*
Age of mother (in years)	1.39	0.64	3.04	0.41
Occupation of mother	0.71	0.31	1.62	0.41
Duration of diabetes in years	1.69	0.74	3.89	0.21
Dietary adherence	0.44	0.18	1.09	0.07
Physical activity per week (in days)	0.950	0.69	1.31	0.75
HbA1c (%)	0.433	0.19	0.97	0.04*

## Discussion

The present study assessed the domain-wise and overall DSQoL among 141 adolescents with T1DM. The mean overall DSQoL score was 66.60 ± 11.30, indicating a moderate level of QoL in the study population. The overall mean DSQoL score observed in this study is comparable to findings from earlier studies using the PedsQL™ Diabetes Module, which have consistently reported moderate QoL levels among pediatric patients with T1DM [3,5,8].

Among the individual domains, Diabetes Management had the highest mean score (69.89 ± 13.45), suggesting relatively better coping with daily management activities such as insulin administration, glucose monitoring, and adherence to treatment routines. Higher mean scores in the Diabetes Management domain may reflect increasing familiarity with diabetes care routines, structured education programs, parental supervision, and access to diabetes care services. By contrast, the Diabetes Symptoms domain had a comparatively lower mean score (63.58 ± 10.79), reflecting a greater negative impact of diabetes-related symptoms such as hypoglycemia, hyperglycemia, fatigue, and physical discomfort on daily life. The relatively lower scores in the Diabetes Symptoms domain are consistent with previous literature, which identifies symptom burden, particularly hypoglycemia, glycemic variability, and physical discomfort, as major contributors to impaired QoL in adolescents with T1DM [4]. Symptom-related distress may persist even in children and adolescents who demonstrate acceptable metabolic control, underscoring the importance of symptom-focused clinical assessment. The wide range of scores observed across domains (minimum 32-36; maximum 90-97) indicates substantial inter-individual variability in perceived QoL, highlighting the heterogeneous experience of living with T1DM among children and adolescents.

Factors including age, occupation of father, duration of diabetes, dietary adherence, physical activity, and glycemic control were found to be associated with DSQoL in univariate analysis, which was supported by many international and Indian studies [5,6,11].

In the multivariable binary logistic regression analysis, age of the child, occupation of the father, and glycemic control emerged as significant independent predictors of DSQoL.

Adolescents aged 14-18 years were significantly more likely to report optimal DSQoL compared to younger children, with nearly threefold higher odds (aOR = 2.70; 95% CI: 1.19-6.16; p = 0.018). This finding is supported by global studies using the PedsQL™ Diabetes Module, which demonstrate that increasing age is associated with better emotional functioning, treatment coping, and self-management skills [3,4]. Indian studies have reported similar age-related trends, with older adolescents showing better psychosocial adjustment and higher QoL scores than younger children [5].

Father’s occupation showed a significant association with DSQoL, with children of fathers in formal employment having more than twice the odds of reporting optimal QoL compared to those in informal employment (aOR = 2.38; 95% CI: 1.04-5.46; p = 0.040). This observation aligns with Indian evidence indicating that socioeconomic stability and parental employment are important determinants of psychological well-being and QoL in children with T1DM [5,11]. International literature similarly highlights socioeconomic context as a key factor influencing pediatric diabetes outcomes and QoL [12].

Good glycemic control was a significant protective predictor of DSQoL. Children with HbA1c <7% had significantly lower odds of impaired QoL (aOR = 0.43; 95% CI: 0.19-0.97; p = 0.043). This finding is strongly supported by global studies reporting an inverse relationship between HbA1c levels and DSQoL, where poor metabolic control is associated with greater treatment burden and emotional distress [13,14,15,16]. Indian studies have similarly demonstrated that better glycemic control is associated with higher QoL scores [6,7].

Other variables, including type of family, maternal age and occupation, duration of diabetes, dietary adherence, and physical activity, did not retain statistical significance in the adjusted model. This suggests that their influence on QoL may be mediated through age, socioeconomic status, and glycemic control, as also suggested in previous Indian and International studies [4,14].

Limitations

This study has certain limitations. First, its cross-sectional design prohibits establishing causal relationships between diabetes-related factors and QoL. Second, being conducted at a single tertiary care center, the findings may not be generalizable to the wider population of adolescents with T1D. Third, QoL was assessed using a self-reported instrument (PedsQL Diabetes Module), which may be subject to recall bias. Finally, potential psychosocial predictors such as family functioning, mental health status, and adolescent self-efficacy were not comprehensively evaluated.

## Conclusions

The results demonstrate that most adolescents withT1DM have a reduced QoL due to their condition. This emphasizes the necessity of regular QoL evaluation as a crucial part of diabetes treatment, in addition to glucose monitoring. In order to lessen the burden of disease and enhance general well-being, interventions should go beyond medical management and include psychological counseling, family-centered support, and adolescent-friendly educational initiatives. Better long-term health outcomes and increased adherence to diabetes treatment may result from early detection and focused care for adolescents with reduced QoL.
